# Sex and Reproduction in the Transmission of Infectious Uveitis

**DOI:** 10.1155/2014/683246

**Published:** 2014-07-01

**Authors:** Janet L. Davis

**Affiliations:** Bascom Palmer Eye Institute, Miller School of Medicine, University of Miami, 900 NW 17th Street, Miami, FL 33136, USA

## Abstract

Current data permit only speculations regarding sex differences in the prevalence of infectious uveitis between women and men because uveitis case surveys do not uniformly report gender data. Differences in prevalence that are reported in the literature could relate to simple differences in the number of women and men at risk for infection or to biological differences between men and women. Compared to other types of uveitis, infectious uveitis may be directly related to occupational exposures or sexual behaviors, which differ between women and men, and may mask actual biological differences in susceptibility to ocular manifestations of the infection and its prognosis. In infectious uveitis for which there is no element of sexual transmission and data is available, prevalence of ocular disease is roughly equal between women and men. Women also have a unique relationship with infectious uveitis in their role as mothers. Vertical transmission of infections such as herpes simplex, toxoplasmosis, and cytomegalovirus can produce severe chorioretinitis in neonates.

## 1. Introduction

Uveitis, especially noninfectious uveitis, is more common in women than in men, in most large surveys, presumably because of the greater frequency of autoimmune diseases in women. The same cannot be presumed to hold true for infectious uveitis. Sex-determined biological variations in type or intensity of response to infections may exist, just as they appear to exist in noninfectious and autoimmune disorders. If anything, the sex differences in infectious uveitis are likely to be greater than in autoimmune disorders because exposure to infections involves behavioral and cultural issues not encountered with the other uveitides.

Differences in sexual behaviors between men and women would predict that uveitis associated with sexually transmissible diseases such as HIV and syphilis would be even more likely to show gender disparities than infections transmitted through environmental and occupational exposures. Monogamy and strict adherence to ideals of chastity and faithfulness in some cultures are important social factors that would likely reduce the prevalence of sexually transmitted infectious diseases in women. Examples that would increase risk of infection among women are sex work and heterosexual transmission from bisexual or promiscuous male partners. Greater exposure increases the risk of infectious uveitis, even though uveitis typically arises in only a small percentage of infected individuals.

It is unknown whether men or women would have greater susceptibility on a biological basis to uveitis associated with infectious diseases. In general, men seem to be more susceptible to infections in multiple species [1]. The development of uveitis may depend on other factors that would skew prevalence toward one sex or the other. For nonsexually transmitted types of infectious uveitis, the difference between male and female prevalence seems to be small indicating that large hormonal influences are unlikely. For sexually transmitted diseases, behavioral factors are likely to overshadow any biological effects related to sex-specific gene expression. Calculation of odds ratios based on proportions of women with sexually transmitted infectious diseases only versus those with both systemic and ocular manifestations would require more detailed data than is currently available.

In addition to unequal transmission of infections predisposing to infectious uveitis, women have additional concerns related to vertical transmission of infections during pregnancy, most commonly those infections included in the TORCH spectrum (toxoplasmosis, cytomegalovirus, and herpes simplex) which can have devastating ocular consequences.

It is the purpose of this review to examine infectious uveitis from the standpoint of its relationship to occupational transmission, sexual transmission, and vertical transmission of pathogens.

## 2. Methods

A Medline search was conducted for peer-reviewed articles in English published from 1990 to 2014 that concerned infectious uveitis qualified by search terms such as prevalence, female, congenital, transmission, and HIV. Additional online resources were consulted for information regarding epidemiologic studies. Primary references were scrutinized for other source publications.

## 3. Results

### 3.1. Occupationally and Environmentally Transmitted Diseases

Occupational exposure to pathogens that have a high penetrance of ocular involvement may display unequal sex ratios as dramatic as those related to sexually transmitted disease.* Leptospira* uveitis in India is often associated with farming or other exposures to animals in rural areas, occupations more likely undertaken by men. Other epidemiological patterns include exposure to ground water in tropical climates and rodents in urban areas, which are more likely to affect the general population including women and children [2]. It is estimated that up to 10% of patients with the systemic disease will have ocular manifestations. The male to female ratio of leptospiral uveitis was 3 : 1 in one study [3]. Uveitic manifestations include hypopyon panuveitis, nonocclusive retinal periphlebitis, and neuroretinitis or papillopathy [2].

Sex differences in brucellar uveitis are another example of differing occupational exposures to animal vectors that result in infectious uveitis. Men aged 20–45 years, engaged in butchering or rendering animal carcasses, seem to be at special risk because of exposure to* B. abortus* or* B. suis* (http://www.who.int/csr/resources/publications/Brucellosis.pdf, accessed 12 April, 2014). In contrast, women in Peru are twice as likely as men to have brucellar uveitis [4]. This is because the manufacture, distribution, or consumption of sheep and goat milk products places more women and children at risk of exposure to a more virulent species,* B. melitensis*, (http://www.who.int/csr/resources/publications/Brucellosis.pdf, accessed 12 April, 2014). In Western Iran, brucellosis is more common in housewives than in farmers [5]; however, in both western and central Iran, the male : female ratio was 2.1 [6]. Ophthalmic manifestations, especially in chronic brucellosis, include posterior uveitis in about 40% of patients and anterior, intermediate, or panuveitis in another 15% each. Corneal and optic nerve inflammations can occur [4].

Hunters who field dress animals may acquire toxoplasmosis and, unlike the other zoonoses or Lyme disease from ticks, then transmit the disease through household exposure to the meat. Viable* T. gondii* was isolated from 17%–29% of white-tailed deer hunted in the United States [7]. Women who assume traditional roles of food preparation can be exposed when handling the meat [8]; a social history should include the possible exposure to infected wild meat. Raw meat from US supermarkets, especially pork, may contain toxoplasma oocysts [7]. In endemic areas of toxoplasmosis in Brazil, the production and ingestion of contaminated sausage may be a factor in the very high prevalence of toxoplasma chorioretinitis in that population. Clustering of toxoplasma seropositivity among all ages and sexes sharing the same household suggests that foodborne transmission is important in endemic areas of Brazil [9]. Differences in prevalence of nonsexually transmitted infectious uveitis between the sexes would depend on the amount, type, and infectivity of the activities to which each sex was typically exposed in their culture. When all persons are exposed mainly through food, an equal sex ratio would be expected.

### 3.2. Sexually Transmitted Diseases

The most striking differences between prevalence in men and women would be expected in diseases that are sexually transmitted because exposure involves a large behavioral component. In 2011 in the USA, there were 8.3 per 100,000 primary and secondary syphilis infections in men versus 1.0 per 100,000 in women. A large imbalance in syphilitic uveitis would also be expected. (http://www.cdc.gov/std/stats11/tables.htm, accessed 01 Sep, 2013, 2:00 PM.) The virulence and persistence of syphilis to the point of causing central nervous system or ocular manifestations is influenced by concomitant HIV infection. Among HIV infected patients, syphilitic uveitis is almost exclusively seen in men; in one meta-analysis, 97 of 101 patients were male [10]. This balance may change as the proportion of HIV-infected women grows relative to men. Among non-HIV infected patients the imbalance in syphilitic uveitis is less striking. A small Chinese case series of 14 non-HIV infected patients with syphilitic uveitis showed only a slight male predominance [11]. Regional factors likely play a role in these results. Syphilis may be more evenly distributed between men and women in China because of fewer men having sex with men or other behavioral factors. Access to care for early treatment or frequency of screening may differ between men and women. Penetrance of syphilis infection in the Chinese population may also be less overall than in the West, resulting in a skewed sample from a very small number of patients; epidemiological information about syphilis in China could not be obtained from online resources. In the United States, the CDC does not separately tally syphilitic uveitis, but only tallies total cases of syphilis in men and women. Conversely, cases of syphilitic uveitis are usually reported without background information regarding the number of cases without ocular disease from the same population and without concurrent controls that have syphilis but not uveitis and might also show gender imbalances from which susceptibility to symptomatic ocular disease could be ascertained. Presumably intraocular involvement is considerably rarer than the systemic infection that causes it in any cultural setting, although the uveitis or optic neuropathy can be most symptomatic manifestation of untreated latent disease [12].

**Figure 1:**
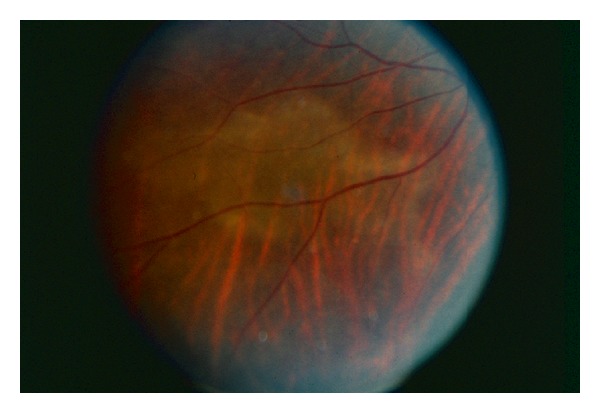
Right eye of a middle-aged married housewife with the placoid variant of syphilitic uveitis. Contact tracing through the health department indicated presumptive infection through her spouse. Preconceptions about the likelihood of syphilitic uveitis should not defer testing of all uveitis patients for exposure.

An international series of syphilitic uveitis of the posterior placoid variant recorded 9 of 60 (15%) of the newly reported and previously published patients to be females [13]. HIV infection was confirmed in 9 of the current and 14 of the historical patients (38%). Among HIV negative patients there was a much higher number of women: 8 of 37 HIV negative patients were women (24.3%) versus 1 of the remaining 23 patients (4.3%). The relatively low prevalence of HIV infection in this series of syphilitic uveitis raises the issue of whether the specific ocular manifestations of syphilitic uveitis, such as the posterior placoid variant, could be influenced by transmission to either a healthy or immunocompromised host, with healthier individuals perhaps more likely to have a limited posterior infection without panuveitis, Figure 1. Understanding the relative influences of immune status, particularly HIV infection, sex, and sexual behavior on syphilitic uveitis would depend on publication of more cases from populations with known seroprevalence of prior syphilitic infection and disease frequencies of symptomatic late manifestations. Prospective studies in sexually transmitted disease clinics are not feasible due to the early treatment of most patients and reduction in their risk of later manifestations of infection, such as uveitis.

Cytomegalovirus retinitis (CMVR) is of particular interest because the virus is sexually transmitted [14] as well as transmitted by body fluids and was the predominant cause of blindness and visual disability among HIV-infected patients prior to the initiation of highly active antiretroviral treatment (HAART) in 1996. Prior to the introduction of HAART, the incidence of AIDS indicator infections was dependent on the degree of immunodeficiency rather than sex [15]. CMVR was not specifically analyzed in this study. Among HIV positive women in the early years of the AIDS epidemic, only those who had nonsexually transmitted HIV such as injection drug-use had an increased risk (odds ratio 1.43) for cytomegalovirus disease [16]. After the introduction of HAART, data from a large multicenter study of the ocular complications of AIDS documented that the percentage of incident cases of CMVR in women more than doubled after the introduction of HAART (35.4% versus 15.3%), a statistically significant change [17]. Demographic differences were attributed to differences in access to care between men and women. Reanalysis of data in 2012 from the same cohort no longer found a difference in the incidence of CMVR between men and women [18]. Globally, testing and treatment for HIV infection is now more readily available to women, which may help reduce risk of opportunistic infections such as CMVR. CMV can also be transmitted through household contacts [19], placing women at special risk if they care for infected young children. In general, unlike syphilis, and independent of HIV status, healthy women are more likely to be infected with CMV than men (OR 1.17 [1.14–1.21]) [19].

### 3.3. Nonsexually Transmitted Diseases

Infectious uveitis caused by nonsexually transmitted pathogens would be predicted to be associated with fewer sex differences. Nonetheless, infectious uveitis of any type is a concern in HIV patients, the sex ratio of which varies according to geographic region therefore variably exposing women. In a large cohort study of HIV-infected individuals, herpes class viruses other than CMV (simplex, zoster), toxoplasmosis,* Cryptococcus*, and atypical* Mycobacterium* had similar prevalence between men and women [20]. Biologic differences related to sex were therefore not apparent in these variably immunocompromised individuals, although case numbers were low. A large series of 111 non-HIV infected Turkish patients, with herpetic iridocyclitis, showed a slight female predominance of 1.2 : 1.0 [21]. In the United States, women are more commonly affected than men with herpes simplex 2 (http://www.cdc.gov/std/Herpes/STDFact-Herpes.htm, accessed 01 Sept, 2013 2:00 PM). A similar situation may have influenced the sex disparity in the Turkish series; patients were not typed as having HSV 1 or 2. A Hawaiian cohort showed no sex imbalance in prevalent cases of herpes zoster ophthalmicus [22]. Chronic anterior uveitis, associated with rubella, herpes, and cytomegalovirus, was slightly more common in men than women in a cross-sectional study of 166 Saudi patients; population seroprevalence of the candidate viruses was not reported [23]. It is unclear whether small differences of this type are due to the prevalence of the primary infection or somehow related to a sex-based susceptibility to the eye disease.

For tuberculosis, there is male predominance although it is among the top three causes of death for women world-wide [24]. Some of this imbalance may be due to the 13% of TB cases that are in HIV-positive individuals; however, most of these are in the African region where the sex balance in HIV infection is more equal than in the European or American regions [24]. In Saudi Arabia, a large survey of uveitis etiologies revealed presumed tuberculous uveitis to be the most common type of uveitis. Male and female prevalence was essentially equal [25], whereas some immunological causes of uveitis were statistically more common (Vogt-Koyanagi-Harada and multiple-sclerosis related) or less common (Behçet) in women than in men. An excellent review of prior publications in TB uveitis from the same group summarizes clinical manifestations, most commonly posterior, panuveitis, or occlusive retinal periphlebitis [26]. Specific sex imbalances are not mentioned.

### 3.4. Vertical Transmission

Women also vertically transmit infections during pregnancy that may cause peri- or postnatal infectious uveitis in their children. A recent series of herpes simplex 2 associated acute retinal necrosis in children identified maternal factors such as birth history with the possibility of direct infection through the birth canal or maternal antibodies in the majority of cases [27]. Congenital syphilis remains relatively common in the United States if considered in the light of the good surveillance and treatment of syphilis during pregnancy. (http://www.cdc.gov/std/stats11/tables/1.htm, accessed 01 Sept, 2013 2:00 PM). The number of ocular infections among the 350 annual cases (about 1 in 10,000 live births) is unknown. Cataract, chorioretinal scarring, and optic neuropathy seem to be rare and do not appear in uveitis surveys. Women with positive treponemal tests but negative nontreponemal tests seem to be at a low risk of transmission of congenital syphilis to their children [28].

Rubella infection currently occurs in less than 1 case per 10,000,000 population in the United States [29]. In Oman where rubella is incident in 0.6 of 1000 live births, bilateral chorioretinitis was the most common manifestation [30]. A historical series from the United Kingdom in 1993 also found bilateral retinopathy to be the most common manifestation of congenital rubella syndrome, although it was not related to vision loss [31]. Interestingly, new diagnoses of Fuchs uveitis syndrome, virologically related to rubella [32], declined in US-born patients after institution of the vaccination program in 1959 whereas the percentage of new Fuchs patients that were foreign born increased [33].

Congenital CMV infection is not specifically related to maternal HIV infection and is more common than congenital HIV. The incidence rate of CMVR in HIV infected children was low in the pre-HAART era (0.5 per 100 person-years) and has fallen further in the post-HAART era [34]. Congenital CMVR is therefore not a specifically HIV-related problem. Among non-HIV infected persons, nonwhite women and those in lower socio-economic groups have higher frequencies of CMV seropositivity and therefore are at greater risk of transmitting CMV prenatally if the primary infection occurs during pregnancy [35]. Unlike syphilis, chorioretinitis or other manifestations affecting the visual pathways are present in almost all of the symptomatic congenital CMV infants, who are 5 to 15% of the total born with serological evidence of congenital disease [36]. Preconception immunity does not protect against transmission to the fetus: about half of children with congenital CMV infection are born to preimmune mothers [37]. The ability to infect a fetus even if the primary maternal infection does not occur in pregnancy may relate to periodic reactivations of lifelong CMV infection, similar to other herpes class viruses such as simplex and zoster. Reinfection with other serovars is also possible.

**Figure 2:**
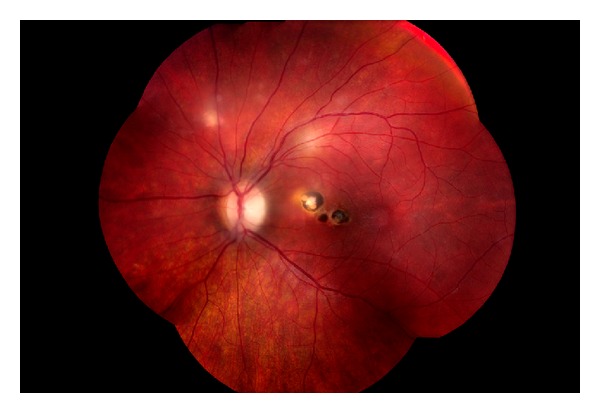
New lesions in the left eye of a child with known congenital toxoplasmosis. The central scars were long-standing. The peripheral lesions occurred in regions previously felt to be normal. Multifocal reactivation is unusual.

The greatest amount of information about vertically transmitted infectious uveitis relates to congenital toxoplasma chorioretinitis. Screening of pregnant women is sometimes undertaken proactively in countries with high frequencies of congenital toxoplasmosis, such as France [38]. This research enabled the establishment of antibiotic regimens for primary prevention of toxoplasma infection in the fetus, if seroconversion occurs during pregnancy. Diagnosis by ocular screening of mothers is not efficient because only 3.8% to 13.7% of mothers of children with congenital toxoplasmosis have chorioretinal lesions consistent with healed toxoplasmosis [39]. As for other infections, screening is performed serologically. Termination of pregnancy is not usually recommended for all women who become infected with toxoplasmosis during the first trimester as only a low percentage of the fetuses will have symptomatic disease [40]. IgG avidity testing can be used to exclude infections that occurred more than 4 months previously despite the persistence of IgM production [41]. Intrauterine sampling can be used to determine if the fetus is infected and ultrasound can detect malformations [42, 43]. Chorioretinitis is the most common ocular lesion in the neonate [44]. If seroconversion is detected in the first trimester, treatment of the mother with antibiotics during pregnancy and the child during infancy can result in good visual outcomes [45]. Postnatal treatment did not prevent the development of new fundus lesions in 34 of 108 (31%) [23–41, 95% C.I.] of affected children [46] but did reduce the incidence of new lesions compared to 18 of 25 (72%) congenitally infected but untreated children [47]. In both groups, about half of the new lesions appeared at age 10 or older, Figure 2. Although in general preimmune women are felt to be incapable of transmitting toxoplasmosis to a fetus, a case has been reported of transmission from a mother who had reactivation of chorioretinitis during pregnancy [48]. As for CMV, reinfection with multiple serovars may be possible.

Lymphocytic choriomeningitis (LCM) virus is a less known congenital infection that produces chorioretinal scarring and vision loss; in one study in Chicago, antibodies against LCM were encountered more frequently in severely retarded children with chorioretinal scars than toxoplasmosis, rubella, CMV, or herpes simplex [49]. In some children chorioretinitis was present but serology did not identify candidate infections indicating the likelihood that other infections can produce fetal ocular infections.

## 4. Conclusion

Women are uniquely affected by infectious uveitides. Vulnerability to HIV from infected partners exposes them to risk of cytomegalovirus retinitis. Syphilitic uveitis is also strongly associated with HIV infection but can also occur in immunocompetent women. Women are also at risk of transmitting infections such as herpes simplex, cytomegalovirus, toxoplasmosis, and lymphocytic choriomeningitis virus, and herpes simplex that can cause chorioretinitis in neonates. Rubella infection produces a relatively benign chorioretinitis that is being eradicated by vaccination programs. Robust screening and treatment programs for vertical transmission of toxoplasmosis have reduced the impact of toxoplasma chorioretinitis on children.
